# Unexpected consequences: Post-traumatic parotid sialocele following facial stab injury: A case report

**DOI:** 10.1016/j.radcr.2024.07.126

**Published:** 2024-08-27

**Authors:** Mehdi Hosseinzadeh, Elaheh Rahimipour, Narges Mirzania, Mahsa Mohammadpour

**Affiliations:** Department of Oral and Maxillofacial Radiology, School of Dentistry, Shahid Beheshti University of Medical Sciences, Tehran, Iran

**Keywords:** Sialography, Sialocele, Parotid gland, Trauma

## Abstract

Parotid sialocele is characterized by a collection of saliva in the soft tissue surrounding the parotid gland. Etiology may be traumatic, iatrogenic, or laceration to the salivary duct or the gland parenchyma itself. We here report the case of a 40-year-old male patient who presented with a primary complaint of swelling on the right side of his face. The patient had a history of facial trauma, having been stabbed in the face, and the swelling developed several months after receiving medical treatment for the injury. Ultrasonography and sialography were used in order to obtain an adequate diagnostic assessment, and the patient underwent parotidectomy surgery. The objective of this article is to raise awareness regarding an uncommon post-traumatic condition involving sialocele formation in the parotid gland following facial cutting trauma.

## Introduction

A sialocele is a subcutaneous cavity filled with saliva, typically arising from trauma or infection of the parotid gland parenchyma, laceration of the parotid duct, or ductal stenosis leading to subsequent dilation [1]. A post-traumatic sialocele is an acquired condition resulting from the extravasation of saliva into the glandular or periglandular tissues due to the disruption of the parotid duct or parenchyma [2,3]. Traumatic causes include sharp penetrating injuries to the oral cavity or face [4,5] and blunt trauma, such as fractures of the zygomatic bone and mandible [5,6].

The diagnosis of sialocele is based on the patient's medical history and clinical evaluation, often involving a history of facial trauma or surgery. Imaging is essential to confirm the diagnosis, assess the extent of ductal injury, and plan appropriate management. In injured patients, the classification of parotid fistulae based on sialographic findings holds prognostic and therapeutic significance, with conservative treatment yielding excellent outcomes [7]. While conventional sialography can be performed, some authors have demonstrated that it may increase the pressure within the sialocele, potentially causing rupture and fistula formation. Nonetheless, sialography remains a primary method for diagnosing and evaluating sialocele [8].

## Case report

A 40-year-old male patient presented with a primary complaint of swelling on the right side of his face. The patient had a history of facial trauma, having been stabbed in the face, and the swelling developed several months after receiving medical treatment for the injury. The patient had no underlying medical conditions and was not on any medications.

**Fig. 1:**
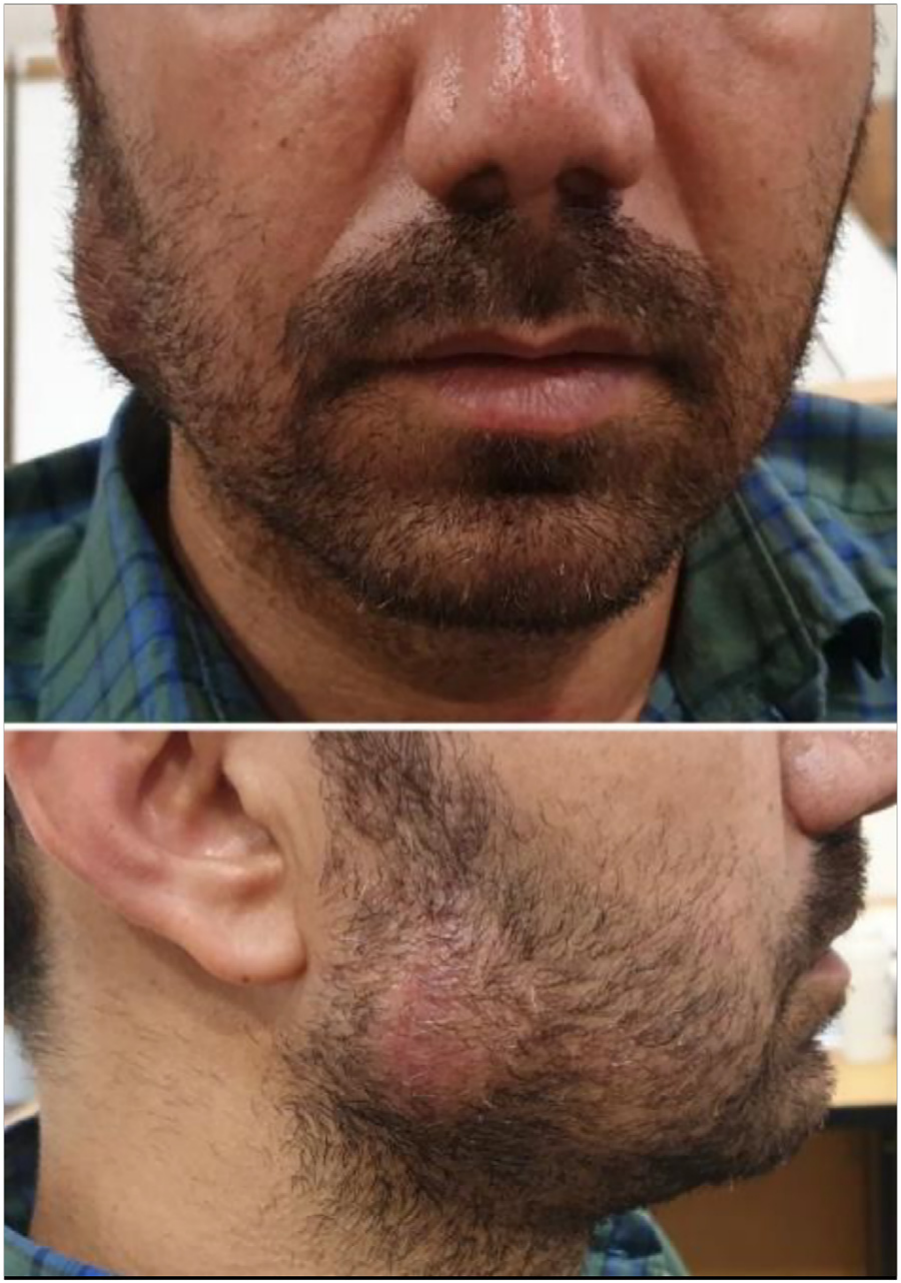
Photograph of the patient is showing a mass extending at the right side of the face. Note the scar on the mass.

During the extra-oral examination, a fluctuant, nontender swelling was palpable in the area of the mandibular angle. Additionally, there was a hypertrophic scar on the face, which was associated with the previous injury sustained in that region (Fig. 1). Aspiration of the lesion revealed 2 mL of clear fluid. Intra-oral examination of the patient appeared normal.

**Fig. 2:**
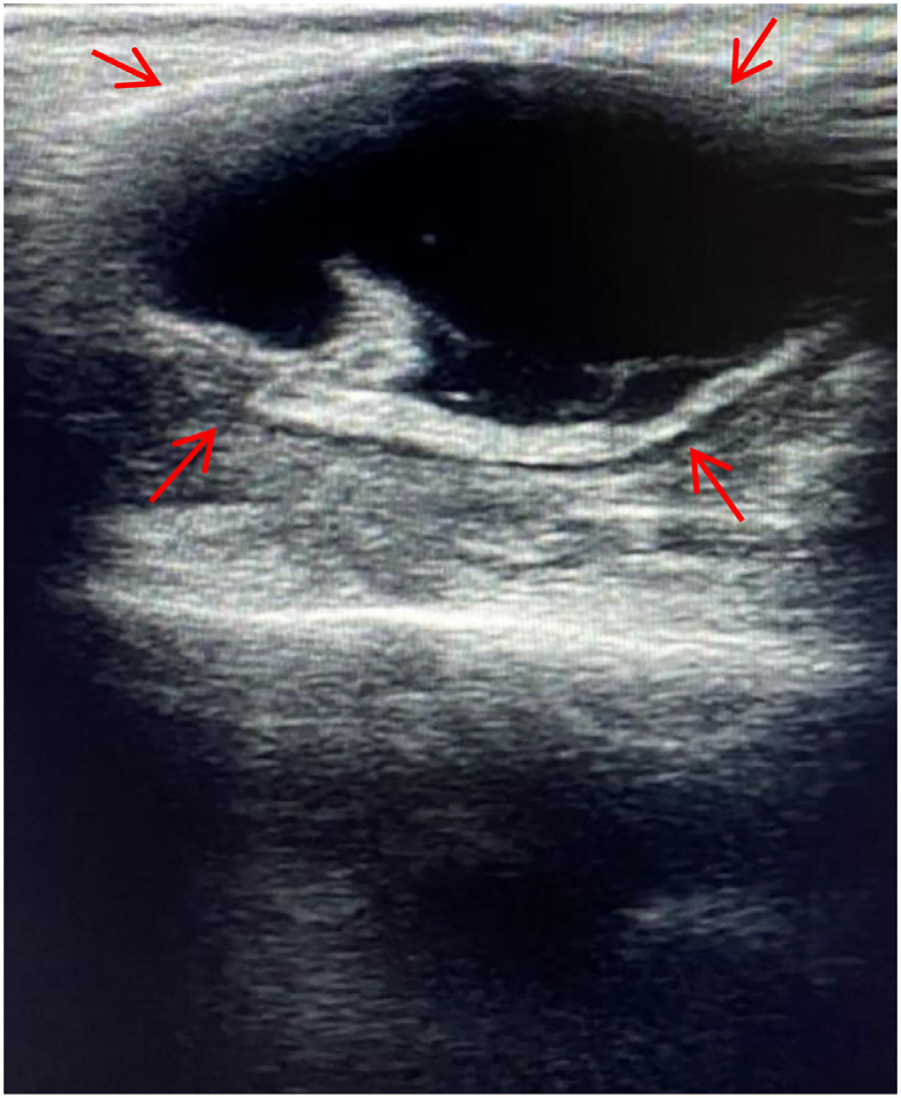
Ultrasonography of the right parotid gland showing a well-defined anechoic lesion in the superficial lobe (indicated by red arrows). No significant vascularity is noted within the lesion on Doppler study.

Ultrasonography of the swollen area confirmed the cystic nature of the lesion, revealing an anechoic lesion within the superficial lobe of the parotid gland, characterized by well-defined margins and accompanied by debris (Fig. 2).

**Fig. 3:**
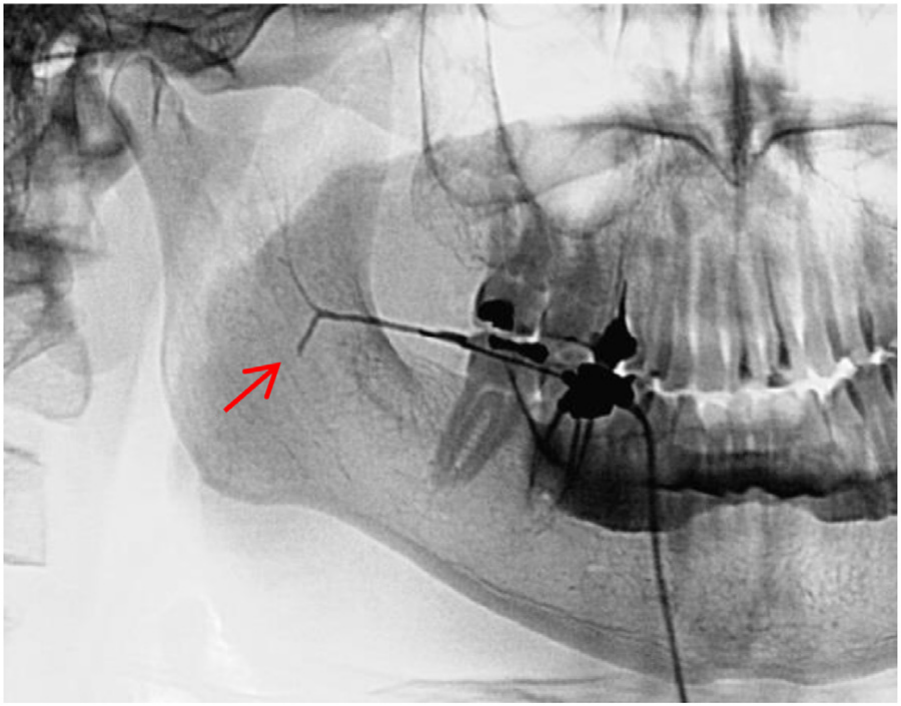
Parenchymal phase of sialography. Note the sharp interruption of the duct marked with a red arrow.

Sialography of the right parotid gland was conducted using Dipigrafin 76% (meglumine diatrizoate 66 g and sodium diatrizoate 10 g) as a contrast medium. During the parenchymal phase, the gland showed incomplete filling of the parenchyma and leakage of the contrast material into the patient's mouth (Fig. 3).

Based on the patient's history, clinical features, investigative findings, and sialographic appearance, the final diagnosis was a rupture in the parotid gland's duct. The rupture led to the formation of fibrous tissue, which subsequently caused ductal obstruction. Additionally, the lesion, resulting from saliva extravasation outside the duct, is most likely identified as a sialocele. The patient was referred to an oral and maxillofacial surgeon and parotidectomy was done.

## Discussion

The pathogenesis of sialocele involves disruption of the gland parenchyma, leading to the leakage of saliva into the surrounding tissues, resulting in the formation of a collection encapsulated by inflammatory pseudocapsule [9]. Parotid duct injury can be attributed to any sort of facial trauma or any iatrogenic cause following surgery of any proximal structure. A common manifestation of this injury results in complete or partial obstruction of duct due to severing leading to pooling of saliva in periductal region [10].

Diagnostic imaging options for ductal stricture include ultrasound, conventional sialography with contrast infusion, magnetic resonance sialography, and sialendoscopy. Among these, sialography stands out as the most sensitive modality for characterizing ductal stenosis, offering detailed information on ductal anatomy, including visualization of the entire ductal system, and assessment of ductal function, such as the clearance rate of infused contrast after sialagogue administration [11].

Sialocele typically manifests following sharp penetrating trauma, with onset usually occurring between the eighth and 14th day after the traumatic event. Conversely, post-traumatic sialocele resulting from blunt trauma tends to develop in a more delayed manner [12].

The management of sialocele has been a subject of debate, with various surgical and nonsurgical approaches proposed in the literature. Nonsurgical options include repeated needle aspiration, compression dressings, radiation therapy at doses ranging from 6 to 20 Gy, and the use of anti-sialogogues such as atropine or probanthine, although their adverse effects limit their utility. Botulinum toxin injection has also been employed in the treatment of parotid sialocele. Surgical modalities include late primary repair or reconstruction of the duct, creation of a controlled internal fistula, superficial or total parotidectomy, parasympathetic denervation, sectioning of the auriculotemporal nerve, and duct ligation. However, many of these procedures are invasive and associated with suboptimal success rates. Another treatment option involves intraoral parotid duct catheterization [13,14]. For the current case under consideration, parotidectomy was performed.

## Conclusion

The objective of this article is to raise awareness regarding an uncommon post-traumatic condition involving sialocele formation in the parotid gland following facial cutting trauma.

## Patient consent

Written, informed consent for publication of this case report was obtained from the patient. The patient has reviewed the case details and has agreed to its publication.
